# Localizing Brain Regions Associated with Female Mate Preference Behavior in a Swordtail

**DOI:** 10.1371/journal.pone.0050355

**Published:** 2012-11-29

**Authors:** Ryan Y. Wong, Mary E. Ramsey, Molly E. Cummings

**Affiliations:** Section of Integrative Biology, University of Texas at Austin, Austin, Texas, United States of America; University of Jyväskylä, Finland

## Abstract

Female mate choice behavior is a critical component of sexual selection, yet identifying the neural basis of this behavior is largely unresolved. Previous studies have implicated sensory processing and hypothalamic brain regions during female mate choice and there is a conserved network of brain regions (Social Behavior Network, SBN) that underlies sexual behaviors. However, we are only beginning to understand the role this network has in pre-copulatory female mate choice. Using *in situ* hybridization, we identify brain regions associated with mate preference in female *Xiphophorus nigrensis*, a swordtail species with a female choice mating system. We measure gene expression in 10 brain regions (linked to sexual behavior, reward, sensory integration or other processes) and find significant correlations between female preference behavior and gene expression in two telencephalic areas associated with reward, learning and multi-sensory processing (medial and lateral zones of the dorsal telencephalon) as well as an SBN region traditionally associated with sexual response (preoptic area). Network analysis shows that these brain regions may also be important in mate preference and that correlated patterns of *neuroserpin* expression between regions co-vary with differential compositions of the mate choice environment. Our results expand the emerging network for female preference from one that focused on sensory processing and midbrain sexual response centers to a more complex coordination involving forebrain areas that integrate primary sensory processing and reward.

## Introduction

Choosing with whom to mate is one of the most important decisions a female makes in her lifetime. While the evolutionary consequences of female mate choice are well documented in a variety of taxa [1], the causal mechanisms are less understood. Typically females have to perceive, integrate, and evaluate multiple cues from at least one male in order to decide which male to copulate with. The majority of studies to date examining proximate mechanisms of female mate choice largely focus on the perceptual stage of this process by studying the peripheral sensory properties [2]–[4], sensory processing centers in the brain [5]–[10], and the influence of hormonal state on female perception of mate cues and mate decision processes [11]–[14]. From this vast body of research, we are beginning to understand how perceptual mechanisms both in the periphery and central nervous system influence a female's behavior during mate choice encounters. However, relatively less is known about the role brain regions beyond those associated with sensory processing play in the mate choice process.

Investigations into other social behaviors such as aggression, parental care, and copulation have focused on a specific network of non-sensory brain regions termed the Social Behavior Network (SBN, [15]). Originally characterized in mammals [15], studies have demonstrated that the SBN is highly conserved and is identifiable in reptiles, birds, amphibians, and teleost fish [16], [17]. While there is evidence that specific nodes of the SBN underlie female reproductive behaviors such as lordosis and copulation [15], [18]–[20], the role that the SBN plays in mate choice is only beginning to be explored [21]. Here we utilize a classic taxa in sexual selection, the swordtail fish (*Xiphophorus nigrensis*), to assess whether brain regions involved in female mate preference extend beyond the SBN and sensory processing regions. We propose that the assessment-based nature of mate preference behavior may also involve brain regions that mediate experience-dependent responses. Hence, we predict that in addition to the SBN we will find brain-behavior correlations in brain regions integrating multiple sensory information, as well as those mediating recall, reward or learning of male phenotypes. To test this, we look at five SBN regions (see Table 1), two representatives of reward circuitry (Dm, Dl, see Table 1) including one associated with learning and memory (Dl) and one associated with multisensory integration (Dm) [22], [23], two additional regions (HV, Pit) selected for their involvement in social behavior or endocrine functions in other species [24], and 1 control brain region (Cb, see Table 1).

**Table 1:** Brain region terminology, putative tetrapod homologue and pathway classification.

	Teleost Region	Putative Tetrapod Homologue	Pathway Classification
Cb	cerebellum	cerebellum	Neither SBN nor Reward
Dl	area dorsolateralis telencephali	pallial hippocampus	Reward
Dm	area dorsomedialis telencephali	basolateral amygdala	Reward
GC	central gray	periaqueductal gray	SBN
HV	hypothalamus ventralis	ventral hypothalamus	Neither SBN nor Reward
Pit	pituitary	pituitary	Neither SBN nor Reward
POA	nucleus preopticus	preoptic nucleus	SBN
TA	nucleus tuberis anterioris	ventromedial hypothalamus	SBN
Vs	ventralis supracommissuralis telencephali	medial amygdala	SBN
Vv	area ventroventralis telencephali	lateral septum	SBN and Reward

*Xiphophorus nigrensis* exhibits a female choice mating system consisting of multiple male phenotypes that differ in body size, ornamentation (e.g. sword and ultraviolet ornamentation), and mating strategy (courting vs. sneak copulation) [25], [26]. In general, females prefer larger sized and courting males, more active males, and those with UV ornamentation [25], [27], [28]. In the wild females might encounter multiple males that vary in ornamentation and mating strategies within a single day, and must presumably evaluate multiple male cues in addition to recognizing (i.e. remember) sneak copulators and courting males. *X. nigrensis* female mate preferences are consistent in both the wild and laboratory conditions [29] and are readily elicited by only visual cues [25]. Therefore, we can manipulate the social encounters of females in the lab and quantify their preference behavior without sexual contact. Hence, this system provides a powerful taxonomic group to explore the neural expression of pre-copulatory mate choice behavior.

The goal of the current study is to identify whether brain regions within or outside the SBN are associated with female *X. nigrensis* mate preference. We do this by localizing expression patterns of two genes previously associated with female mate preference contexts in *X. nigrensis* [30]–[32] – *egr-1* (an immediate early gene and transcription factor) and *neuroserpin* (a serine protease inhibitor). We use both *egr-1* and *neuroserpin* to provide two lines of evidence for a brain region's involvement in female mate preference. We focus on these two genes because 1) they have context-specific associations with mate choice conditions [30], [32], 2) whole-brain expression for both genes is correlated with preference behavior in male-exposed females [30]–[32], and 3) both are involved in synaptic plasticity processes [33]–[37]. Neural activity markers are often used to identify brain regions involved in social behaviors [10], [35], [38]–[41]), particularly members of the immediate early gene family, such as *egr-1. Egr-1* is rapidly up-regulated in response to extracellular stimuli and peaks in mRNA expression approximately 30 minutes post-stimulation [35], [38], [39]. *Egr-1* can also directly regulate *neuroserpin* expression in cell cultures [34]. *Neuroserpin* is an extracellular serine protease inhibitor implicated in modulating synaptogenesis and synaptic plasticity [36], [37], [42], [43], and may modulate exploratory behavior in mice [44]. Further, *neuroserpin* exhibits contrasting patterns of expression in related teleost species with mate choice (positive) versus mate coercive (negative) mating systems [31].

In this study we identify brain regions associated with female *X. nigrensis* mate preference by analyzing changes in *egr-1* and *neuroserpin* expression within 10 brain regions and subsequently discuss their potential functions in the mate preference context. We utilize the multiple swordtail male phenotypes to create diverse social conditions and assess if gene expression patterns are reflective of exposure to different compositions of male pairs. Our three male-exposure contexts represent a presumed gradient in both sensory arousal and mate choice complexity with the LL (two large males) treatment context representing high sensory arousal and a relatively complex mate preference environment (two attractive males where females must discriminate males based on multiple characteristics), whereas LS (large and small male) and SS (two small males) treatment contexts may elicit lower sensory arousal while representing a simple and minimum mate preference environment, respectively. In addition to the three mate choice pairings, we also exposed females to a non-choice female only condition (FF), and an asocial context. In two separate experiments we expose females to one of the social conditions for 30 minutes in a dichotomous choice assay, and for each female we quantify (i) preference and behavioral displays, and (ii) *egr-1* (Experiment 1) or *neuroserpin* (Experiment 2) expression in 10 brain regions. We present evidence that in *X. nigrensis*, female mate preference involves brain regions extending beyond the SBN and includes forebrain regions involved in multi-sensory processing and learning and memory.

## Methods

### Ethics Statement

All experimental procedures were approved by the Institutional Animal Care and Use Committee at the University of Texas at Austin (protocol #07110101).

### Paradigm

All experiments were conducted with sexually mature wild caught or progeny of wild caught female *X. nigrensis* maintained at University of Texas Brackenridge Field Laboratories in Austin, Texas. Immediately prior to the behavior trials, as a control we measured a proxy for circulating estradiol to account for potential influences of estrogen on behavior or localized gene expression patterns through a non-invasive waterborne assay (see below). We followed established behavioral measurements, dichotomous choice paradigm, and natural lighting conditions to assess preference in this species [30]. Briefly, the behavioral testing arena (120 cm×30 cm×48 cm aquarium) was divided into five 24 cm zones. One zone at each end of the tank contained stimuli fish behind a barrier. The three remaining subsections are open to the focal female and consisted of a middle “neutral” zone with an “association” zone adjacent to each stimulus. Females were acclimated for 5 minutes in the center (neutral region) of the experimental tank and allowed to interact with either end stimuli (behind UV-transparent plexiglass partitions) for 30 minutes, with stimuli switched after 15 minutes to disassociate female preference for a specific stimulus from side bias.

For each behavioral trial we recorded (i) the number of female glides (a display wherein the female initially orients towards the stimulus, turns and swims away, but then returns to the initial stimulus-facing position; glides are considered a proxy for receptivity and can precede copulatory events in *X. nigrensis* and related species [45]–[47]), (ii) the overall locomotor activity of each female by counting transits (number of times a female swims into a central neutral zone in the tank), and (iii) association bias. Association bias is defined as the proportion of time spent with stimulus *a* (i.e. in association zone adjacent to stimulus *a*) where time spent with stimulus *a* > stimulus *b*. Since females can have similar association biases but vary in the frequency of behaviors (e.g. glides and transits), we calculate a composite preference score that encompasses both time and behavior (preference score (PS)  =  association bias + log [(1 + receptivity displays towards the biased stimulus)/total transits]) as in [30], [32], [48]. As the PS involves a log transformation of our behavioral measures, more positive PS indicates the female showed both a relatively higher bias in association time and glides toward one stimulus (normalized by general locomotor activity); whereas more negative PS indicates the female generally showed relatively little bias in association time and/or behavior.

Females in Experiment 1 (*egr-1* quantification) were subjected to one of three conditions: a mate choice context (one large and small male, LS, n = 10), two size-matched females (FF, n = 10), or to an asocial control (AA, n = 10) wherein the focal female was placed in the experimental tank without any stimuli at either end. All size-matched stimuli differed by no more than 1 mm standard length. We conducted Experiment 2 (*neuroserpin* quantification) independently to determine if we saw similar localization patterns and preference behavior associations between a general activity marker (*egr-1*) versus our more context-specific marker (*neuroserpin*). As we previously demonstrated an association between mate preference and whole-brain *neuroserpin* expression [30], [31], we expanded the social exposure paradigm to include presumed levels of complexity (see below). Females in Experiment 2 (*neuroserpin* quantification) were subjected to one of five conditions: two size-matched large males (LL, n = 10) with one male behind a UV pass barrier and the other behind a UV blocking filter, one large and small male (LS, n = 13), two size-matched small males (SS, n = 7), two size-matched females (FF, n = 12), or collected from their home tank (HT, n = 6), a treatment serving as an asocial control context (females are housed in isolation). Home tank females underwent the same pre-testing estradiol measurements as females exposed to the other contexts but were then returned to their home tanks for 30 min. prior to sacrifice. We selected our three male-exposure conditions to represent a gradient in mate choice complexity with the LL treatment context representing a relatively complex mate preference environment (two preferred phenotypes varying in UV ornamentation), and the LS and SS treatment contexts representing a simple and minimum mate preference environment, respectively. Females can both see and prefer males with UV ornamentation [28]. Therefore we used UV pass and blocking filters in the LL trials to allow females to discriminate between two attractive males by a secondary sexual characteristic other than size in this more complex condition.

Females were isolated at least two weeks before behavioral testing to ensure sexual motivation. Each female was pre-tested twice with large/small stimuli prior to context assignment to ensure similar baseline preference responses across experimental contexts. Females in Experiment 1 (large/small, female/female, and asocial conditions) or Experiment 2 (large/large, large/small, small/small, female/female, home tank condition) showed no significant differences in pre-test preference trials (*egr-1* ANOVA, PS: *p* = 0.122; *neuroserpin*, ANOVA, PS: *p* = 0.992).

We identified “high” performing (> median) females for each behavior of interest (preference score, transits, or glides) and compared their gene expression in each brain region (see below) with females identified as “low” performing (< median). For context specific comparisons, we examined the relationship between gene expression and behavior in each region for females exposed to males (large/large, large/small, and small/small) relative to female-exposed females (FF). We subsequently examined the unique covariation patterns between brain regions for each male-exposed environment (large/large, large/small, or small/small).

### Estradiol Measurements

We quantified estradiol levels for all females through a non-invasive waterborne assay following an established protocol for teleosts [49]–[51] and validated in our focal species [48]. Briefly, females were placed in a 250 mL glass beaker containing 150 mL of reservoir water (treated tap water used for home and experimental tank) for one hour prior to behavior trials. Estradiol was extracted from the water using C18 Solid Phase Extraction columns (Sep-Pak Plus C18 cartridge 55–105 lm; Waters Corporation, Milford, MA) and measured on a Correlate-EIA 17β-estradiol Enzyme Immunoassay Kit (Assay Designs) according to manufacturer's protocol. Hormone samples were run on three 96-well EIA assay plates: inter-assay CV was 6.5% and intra-assay CV was 1.9%.

### Tissue Processing and *in situ* hybridization

Females in each experiment were decapitated within 30 seconds of the end of the behavior trial and brains were frozen on dry ice. We stored tissue at −80°C until sectioning at 16 µm onto serial series. Tissue fixation parameters, probe synthesis, and *in situ* hybridization conditions were modified from established protocols [8], [52]. For Experiment 1 (*egr-1* quantification), we used only a digoxigenin (DIG)-labeled probe. For Experiment 2 (*neuroserpin* quantification), we used DIG-labeled probe for one series and S35-labeled probe for another series. Each series was processed simultaneously to minimize technical variation. Sense riboprobes showed negligible to no expression (Figure S1). Please see Methods S1 for detailed process parameters.

### Gene expression quantification

Using a *X. helleri* brain atlas for reference and terminology [53], we identified and quantified DIG-labeled riboprobe expression in 10 brain regions (Table 1). These brain regions include putative teleost homologs [16], [17], [54], [55] for nodes in the social behavior network (SBN [15], [16]), reward system (the mesolimbic dopaminergic reward pathway [56]), and other regions. While the tetrapod homology of some teleost brain regions are difficult to determine due to different neural developmental trajectories [55], in the current study we follow designations established from other studies focusing on homologies [16], [17], [55]. After tissue processing final sample sizes for Experiment 1 (*egr-1* quantification) were: large/small, n = 6; female/female, n = 7; asocial, n = 10. For Experiment 2 (*neuroserpin* quantification) final sample sizes were: large/large, n = 10; large/small, n = 10; small/small, n = 5; female/female, n = 9; home tank, n = 5.

#### Digoxigenin quantification

We quantified gene expression by measuring the optical density (OD) of the digoxigenin labeled probes, which has been established as a semi-quantitative measure of gene expression in other systems [57]–[60]. For each slide, we normalized the mean intensity of all measures to the background (mean intensity of slide not containing tissue), which produced a value for the fractional transmittance of the brain region in each section. Fractional transmittance was mathematically converted to optical density by the equation OD  = 2-log(Fractional Transmittance), which was derived specifically for the imaging setup (Nikon Eclipse 80i) in our laboratory using neutral density filters 0, 8 and 32. Using NIS Elements image analysis software (Nikon), we measured the OD of *egr-1* and *neuroserpin* expression across individuals from a standardized portion of each brain region (ranging from 1737–29152 μm^2^ depending on size of the brain region of interest, please see Methods S1 for additional details).

#### DIG validation with S-35 riboprobe

For one brain region, Dm, we manually counted the number of S35-labeled cells expressing *neuroserpin* (containing at least one silver grain) to compare with the optical density measures of DIG-labeled *neuroserpin* expression. To quantify the number of *neuroserpin* positive cells for each individual, we averaged the number of cells counted from three consecutive sections (each section spans 48 μm apart). For each section we collected images of two non-overlapping fields of Dm at 100X (each field measured 12124 µm^2^) modifying a previously established protocol [61]. For each unique field, two images were taken: one under brightfield where we focused on cell resolution and the second image taken under darkfield where we focused on silver grain resolution. The counting image was created by superimposing the two original images using Photoshop CS4 (Adobe Systems). Dm images for all individuals were counted by three observers blind to the treatment using Photoshop CS4. Total number of cells did not differ by treatment condition (F = 0.678, p = 0.572).

### Statistics

All statistics were performed in SPSS (ver. 18) and the network statistics were conducted using Ucinet [62]. We used a t-test to examine context-wide behavioral and gene expression differences between individuals expressing high versus low behaviors in each treatment condition and a Benjamini-Hochberg correction [63] for multiple hypothesis testing. To assess relationships between individual variation of preference behavior, gene expression, or estradiol levels we used Pearson's correlation when the data was normal and Spearman correlation when data was non-normal (glides and transits) and corrected for multiple hypothesis testing as above. We calculated effect sizes and conducted a post-hoc power analysis to calculate achieved power (1 – β error probability) using G*Power 3.1 computer software [64]. As the effect size for correlation analyses is the absolute value of the correlation coefficient, we just report the correlation coefficient for simplicity. For the correlation analyses, we designate “high”, “medium”, and “low” effect size boundaries as 0.5, 0.3, and 0.1, respectively, as in [65], [66]. For significant correlations between gene expression and preference score, we additionally ran a randomization test on correlation coefficients with replacement 10^5^ times using freeware provided by Dr. David C. Howell (http://www.uvm.edu/~dhowell/StatPages/Resampling/Resampling.html#Return1). This process allows us to examine the probability that the observed coefficient correlations were due to chance. Randomization with replacement holds the behavioral measure constant and randomly pairs the OD of a brain region to obtain a correlation coefficient. After multiple runs (10^5^), we generated a distribution of correlation coefficients for each brain region and behavioral measure. By comparing the observed correlation coefficient against the generated distribution, we rejected the null hypothesis that *r* = 0 when the observed value had less than 5% probability of occurring.

We directly compared correlation coefficients by doing a Fisher r-to-z transformation and then use a Z-test to assess brain region expression consistency between *egr-1* and *neuroserpin*. Due to uneven sample sizes across experiments we calculated effect sizes and achieved power as above when analyzing consistency of expression between experiments. We calculate effect size (q) following standard methodology (difference between the two Fisher-*z*-transformed correlation coefficients) and designate “high”, “medium”, and “low” effect size boundaries as 0.5, 0.3, and 0.1, respectively, as in [65]. We considered consistent expression across both experiments if we observed non-significant differences between the correlation coefficients across the two experiments for the male-exposed and female-exposed environments.

To begin to identify and characterize a network of brain regions associated with female mate preference, we utilized network analyses [67], [68]. Specifically we examined coordinated patterns (i.e. pairwise correlations) of *neuroserpin* expression across all brain regions, by converting all Benjamini-Hochberg corrected correlations of *neuroserpin* expression between regions into binary values in an association matrix (1 =  significant correlation, 0 =  non-significant). We then analyzed (*i*) the degree centrality of brain regions in each treatment context (large/large, large/small, small/small, female/female, home tank), and (*ii*) the density of these networks. Due to small sample size, we did not analyze *egr-1* network expression patterns.

Degree centrality is a way to assess how connected a node is in a network [67], and here we evaluate it by assessing the number of significant correlations between focal brain regions. The assumption is that a brain region with a high number of correlations with other regions may have a more central role in preference dynamics. Degree centralities for each node in each network were calculated in Ucinet and then compared to other nodes in the same network using a Wilcoxon Rank Sum test in SPSS. For each exposure condition, we calculated effect size and achieved power as above. We used an established formula to calculate effect size (d) for nonparametric analyses (difference between the means divided by the standard deviation [65]). We designate “high”, “medium”, and “low” effect size boundaries as 0.8, 0.5, and 0.2, respectively, as in [65].

We also evaluated network density [67] to assess the complexity of the *neuroserpin* expression response during preference using a t-test. Density is evaluated as the number of unique correlations in each of the male exposed contexts (large/large, large/small, small/small) by removing overlapping correlations found in the controls (FF or HT).

## Results

### Female preference

**Figure 1:**
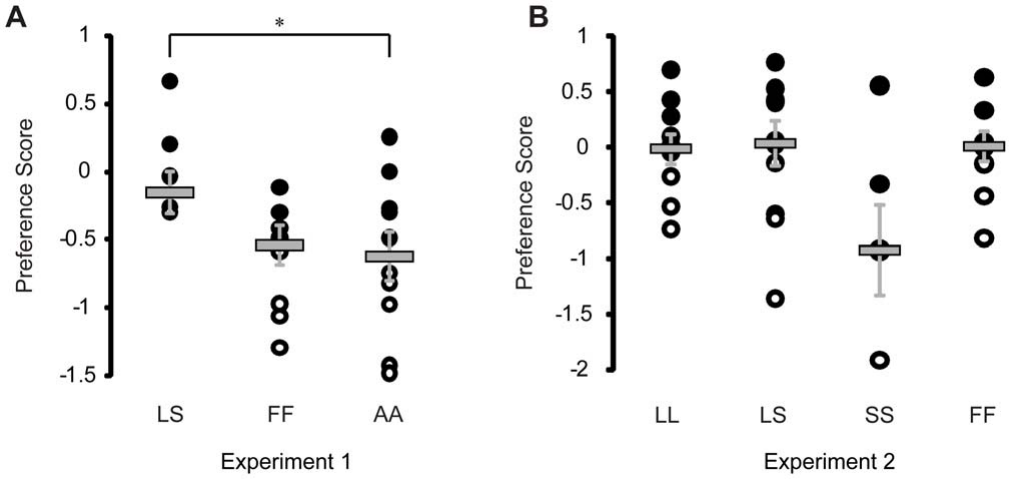
Female preference behavior. Behavioral preference scores for each individual in (a) Experiment 1 (*egr-1* quantification) and (b) Experiment 2 (*neuroserpin* quantification) by treatment context. Gray horizontal line is the median with standard error. Black and white circles represent high (> median) and low (< median) preference score females, respectively. *, p<0.01.

As reported in previous studies [27], [30], [32], [45], females preferred to associate with large males over small males in both Experiment 1 (*egr-1* quantification, large males: 955.5±109.6 sec, small males: 578±116.4 sec, t = 2.3, n = 6, p = 0.039) and Experiment 2 (*neuroserpin* quantification, mean association time ± SE with large males: 1160±78.1 sec, small males: 373.6±50.8 sec, t = 8.4, n = 10, p = 1.1 * 10^−7^) experiments. In Experiment 1 (*egr-1* quantification), females exposed to an empty stimulus environment (AA) displayed a tendency for a side bias (left side association time: 571.8±75.9 sec, right side: 790.7±76 sec, t = −2.0, n = 10, p = 0.056). Females exposed to large/small (LS) conditions in Experiment 1 (*egr-1* quantification) had significantly higher preference scores than females exposed to the asocial conditions (AA, t = 2.3, p = 0.037, Figure 1A), while females in all social exposure contexts of Experiment 2 (*neuroserpin* quantification) exhibited similar ranges of preference scores (F = 1.92, p = 0.14, Figure 1B).

### DIG quantification validation

We conducted *in situ* hybridization of *neuroserpin* expression (Experiment 2) on 39 females using both non-radioactive (digoxigenin, DIG) and radioactive (S35) methods on serial sections. The S35-labeled riboprobes provided validation of the DIG-labeled approach as evidenced by (*i*) a significant positive correlation between the two quantification methods (r = 0.351, p = 0.008, Figure S2A), (*ii*) consistent context-specific *neuroserpin* expression patterns in Dm by both methods for high (> median) relative to low (< median) performing females for preference score, glides and transits (Table S1), and (*iii*) significant correlations between *neuroserpin* expression in Dm and preference score for male-exposed females in both approaches (Figure S2B).

### Localized gene expression and preference behavior

**Figure 2:**
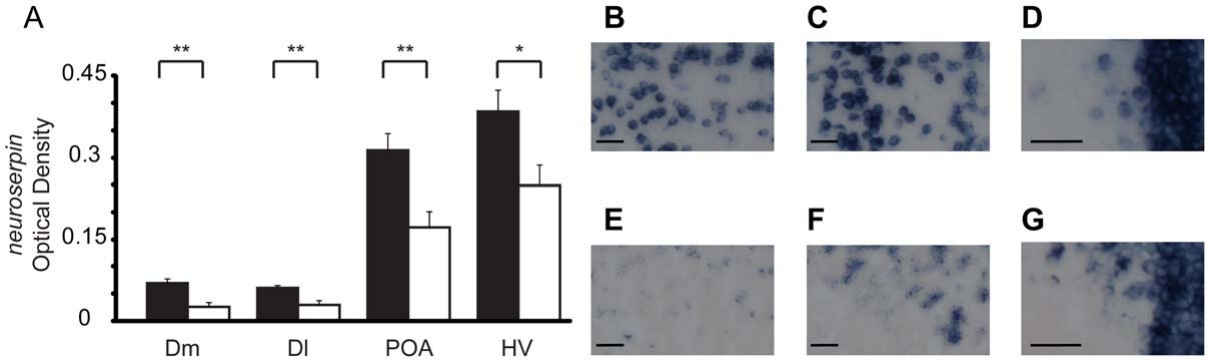
*Neuroserpin* expression in male-exposed females. (A) Significant differences in *neuroserpin* expression of male-exposed females (large/large, large/small, small/small) between groups of high (black) and low (white) preference score females measured in Dm, Dl, POA, and HV. Bars represent standard error. **, p<0.01; *, p<0.05. (B–D) are representative images of a high-preference female (preference score  = 0.4) for Dm, Dl, POA, respectively. (E–F) are representative images of a low-preference female (preference score  = −0.91) for Dm, Dl, POA, respectively. Scale bar is 25 microns.

To quantify DIG-labeled gene expression in 10 different regions we measured the optical density (OD) within each region (see methods). There were no significant across treatment differences in either *egr-1* or *neuroserpin* expression for any brain region after correcting for multiple comparisons (Figure S3). Within exposure context, however, there were clear differences in *neuroserpin* expression between females expressing a high versus low preference score (male exposed or female/female (FF)) within three forebrain (Dm, Dl, POA) and one midbrain (HV) region (Figure 2, Table S2). Male-exposed females (small/small (SS), large/small, and large/large (LL)) but not FF females (Table S2) with high preference scores had significantly higher *neuroserpin* expression than low preference score females in each of these brain regions (Dm, t = 3.284, p = 0.003; Dl, t = 2.91, p = 0.008; POA, t = 3.292, p = 0.003; HV, t = 2.489, p = 0.021, Figure 2, Table S2). The difference in *neuroserpin* OD in HV was not significant after a multiple hypothesis correction.

Using S35-labeled *neuroserpin* riboprobes, we measured expression in Dm and found a significantly greater number of *neuroserpin* positive cells in high preference females over females displaying low preference (mean ± SE: high preference score  = 309.56±22.85, low preference score  = 213.89±18.04, t = 2.079, p = 0.003) in only male-exposed females (Table S1). There were no significant differences in *neuroserpin* expression with either DIG or S35 labeled riboprobes in any context (male exposed or FF) between high versus low performing females for glides and transits (Tables S1 & S3). Due to small final sample sizes we did not analyze *egr-1* expression differences between high versus low preference females.

### Individual variation in gene expression and behavior (region and context specificity)

**Figure 3:**
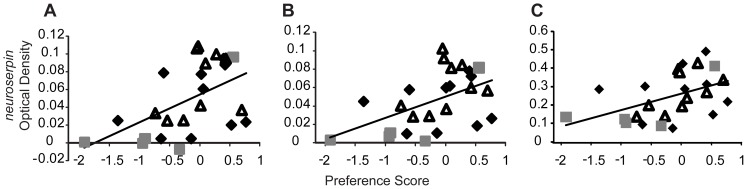
Individual variation of preference score and *neuroserpin* expression. Significant correlations between individual variation in preference score and *neuroserpin* expression in (a) Dm, (b) Dl, and (c) POA. Triangles, diamonds, and squares represent LL, LS, and SS exposed females, respectively.

**Figure 4:**
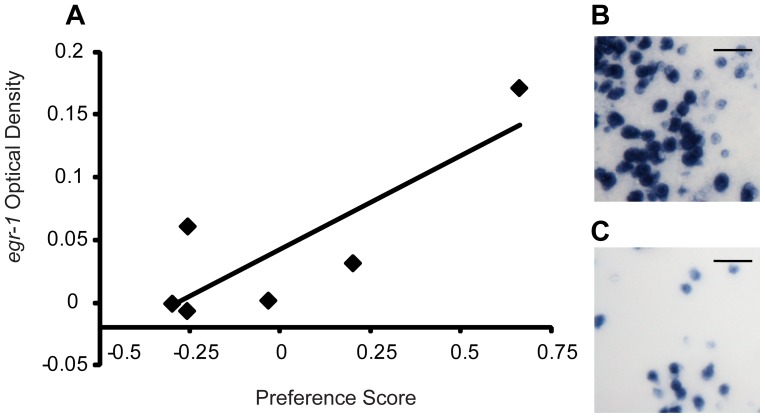
Individual variation of preference score and *egr-1* expression. (a) Significant correlation between individual variation in preference score and *egr-1* expression in Dm. Representative images of *egr-1* expression in Dm for two individuals with a preference score of (B) 0.66 and (C) −0.26. Scale bar is 25 microns.

Of the four brain regions that showed differences in *neuroserpin* OD between high and low preference females, three exhibited significant positive correlations between individual variation of female preference score and *neuroserpin* expression in male-exposed females only: Dm (n = 25, r = 0.522, p = 0.007, Figure 3a), Dl (n = 25, r = 0.501, p = 0.011, Figure 3b), and POA (n = 25, r = 0.479, p = 0.015, Figure 3c) but not HV (n = 25, r = 0.368, p = 0.07). Randomization tests show that the relationships seen between *neuroserpin* expression and preference score in Dm, Dl and POA are not likely due to chance (p<0.02, Table 2). We obtained similar results when quantifying preference score and *neuroserpin* Dm expression with the S35 quantification method (n = 25, r = 0.405, p = 0.049, Figure S2B). *Egr-1* expression was correlated with preference score in male-exposed females in Dm (n = 6, r = 0.829, p = 0.041, Figure 4), a trend in Dl (n = 6, r = 0.797, p = 0.057) but not in POA (n = 6, r = −0.003, p = 0.994), while exhibiting no correlation with mate preference in any other context (Table S4). Randomization test indicate that the relationship between *egr-1* expression and preference score in Dm is also not likely due to chance (p<0.05, Table 2). While we observed a significant correlation between Pit *neuroserpin* expression and preference score (Table 2), we did not observe expression differences in this region between high and low PS females (Table S2). These relationships were mate preference-specific, as neither *egr-1* nor *neuroserpin* expression were significantly correlated with other behaviors (transits or glides) in Dm, Dl, or POA in any condition (Table S5). To assess whether these relationships were driven by the size asymmetry in the large/small context, we removed the large/small context from the analyses and observed that high preference score females in the size-matched male conditions still had significantly higher *neuroserpin* expression than low preference score females in the candidate regions (LL & SS, Dm: t = 5.5 p = 0.0001; Dl: t = 5.6 p = 0.0001; POA: t = 5.1, p = 0.0002), as well as significant correlations between preference score and *neuroserpin* expression (Dm: n = 15, r = 0.678, p = 0.005; Dl: n = 15, r = 0.7, p = 0.003; POA: n = 15, r = 0.7, p = 0.003). In contrast, we do not see a significant correlation between preference score and *neuroserpin* expression in the same brain regions in the size matched female/female context. Finally, there were no significant correlations between circulating estradiol levels and preference score, glides, transits, or gene expression in any brain region for any treatment context after correcting for multiple hypothesis testing (Table S6). This suggests that differences in circulating estradiol levels between individuals are an unlikely explanation for our observed gene expression patterns with mate preference behavior.

**Table 2:** Gene expression (*egr-1, neuroserpin*) correlated with preference score in each of the 10 brain regions (first column) within and between experiments for female exposed to males or females.

	Male Exposed LS + (LL, SS) (if present)				Female Exposed (FF)			
	Experiment 1 (*egr-1*) N = 6	Experiment 2 (*neuroserpin*) N = 25)	Test for significant differences in *r* between experiments		Experiment 1 (*egr-1*) N = 6	Experiment 2 (*neuroserpin*) N = 25	Test for significant differences in *r* between experiments	
	*r* (p-value, achieved power)	*r* (p-value, achieved power)	Z-score (effect size)	p-value (achieved power)	*r* (p-value, achieved power)	*r* (p-value, achieved power)	Z-score (effect size)	p-value (achieved power)
Dm	0.829 (0.041*, 0.58)	0.522 (0.007*, 0.51)	0.98 (0.6)	0.32 (0.52)	0.301 (0.512^ns^, 0.62)	0.24 (0.535^ns^, 0.62)	0.1 (0.07)	0.92 (0.92)
Dl	0.797 (0.057^ns^, 0.58)	0.501 (0.011*, 0.52)	0.88 (0.54)	0.37 (0.53)	0329 (0.471^ns^, 0.6)	0.184 (0.636^ns^, 0.52)	0.24 (0.16)	0.81 (0.82)
Cb	0.192 (0.68^ns^, 0.71)	0.296 (0.151^ns^, 0.51)	−0.2 (0.11)	0.84 (0.84)	−0.257 (0.539^ns^, 0.62)	−0.277 (0.471^ns^, 0.59)	0.04 (0.02)	0.96 (0.96)
GC	0.023 (0.971^ns^, 0.97)	0.187 (0.37^ns^, 0.54)	−0.31 (0.17)	0.75 (0.76)	0.147 (0.754^ns^, 0.77)	0.108 (0.783^ns^, 0.84)	0.06 (0.03)	0.95 (0.95)
Pit	−0.125 (0.773^ns^, 0.78)	0.439 (0.032*, 0.52)	−1.09 (0.6)	0.27 (0.51)	−0.193 (0.646^ns^, 0.69)	0.694 (0.038*, 0.96)	−1.74 (1.05)	0.08 (0.49)
POA	−0.003 (0.995^ns^, 0.99)	0.479 (0.015*, 0.52)	−0.85 (0.52)	0.39 (0.54)	−0.195 (0.711^ns^, 0.74)	−0.309 (0.419^ns^, 0.57)	0.17 (0.12)	0.86 (0.86)
TA	−0.278 (0.546^ns^, 0.63)	0.219 (0.305^ns^, 0.53)	−0.93 (0.51)	0.35 (0.53)	0.045 (0.916^ns^, 0.92)	0.049 (0.901^ns^, 0.9)	−0.01 (0.004)	0.99 (0.99)
HV	0.144 (0.758^ns^, 0.77)	0.368 (0.07^ns^, 0.51)	−0.44 (0.24)	0.65 (0.68)	−0.202 (0.632^ns^, 0.68)	0.043 (0.913^ns^, 0.91)	−0.41 (0.25)	0.68 (0.7)
Vs	−0.48 (0.929^ns^, 0.96)	0.217 (0.297^ns^, 0.53)	−1.21 (0.74)	0.22 (0.5)	0.806 (0.053*, 0.58)	−0.152 (0.719^ns^, 0.74)	1.74 (1.27)	0.08 (0.49)
Vv	0.333 (0.519^ns^, 0.62)	0.161 (0.452^ns^, 0.57)	0.3 (0.18)	0.76 (0.88)	0.403 (0.37^ns^, 0.57)	−0.425 (0.294^ns^, 0.55)	1.31 (0.88)	0.19 (0.51)

Columns in Experiment 1 and Experiment 2 shows within experiment correlation analyses between preference score and gene expression in designated brain region.* indicates significance remains following randomization procedure; ^ns^ indicates significance did not remain following randomization procedures. Z-scores are reported along with effect size (q).

### *Egr-1* and *neuroserpin* regional expression consistency related to behavior

*Egr-1* and *neuroserpin* were expressed in all examined regions in all contexts (Figure S3). We found no significant differences in correlations in region-specific gene expression and preference score across experiments, although our effect size was relatively low for detecting differences in Cb, GC, and Vv brain regions (Table 2). For the male-exposed environments, both *egr-1* and *neuroserpin* showed consistent expression patterns, including consistent positive correlations within Dm and Dl (Table 2). Similarly, both genes showed consistent patterns in the female exposed environments, for all brain regions examined in the current study (Table 2). Effect size calculations showed that overall we achieved medium to high effect sizes in the male (average effect size: 0.421±0.22) and female (average effect size: 0.385±0.48) exposed contexts (Table 2).

### Context-specific expression networks

Looking at within network dynamics, candidate regions associated with mate preference (Dm, Dl, and POA) had a significantly higher degree centrality relative to the other seven brain regions in females exposed to males (Z = 2.44, p = 0.015 (large/large); Z = 2.34, p = 0.019, (large/small); and Z = 2.59, p = 0.009, (small/small); Table 3) whereas females exposed to females (p = 0.55) or asocial conditions (p = 0.35) showed no significant difference. For instance, in the large/large context, the degree centrality of Dm (0.33), Dl (0.44) and POA (0.57) was 3–5 times greater than the average of the other brain regions (mean degree centrality  = 0.158; Table 3). Post-hoc power analyses show that overall the effect size was high (Table 3).

**Table:** **Table 3.** Degree centrality of *neuroserpin* by social context for each brain region, in each treatment group for females used to localize *neuroserpin*.

	Dm	Dl	Cb	GC	Pit	POA	TA	HV	Vs	Vv	Average degree centrality of candidate regions ± standard error	Average degree centrality of other regions ± standard error	Wilcoxon rank sum Z-score (effect size)	p-value (achieved power)
LL	0.33	0.44	0.11	0.11	0.11	0.56	0.11	0.11	0.22	0.33	0.44±0.06	0.16±0.03	2.44 (2.85)	0.015 (0.78)
LS	0.33	0.22	0.11	0.11	0	0.22	0.11	0.11	0.11	0.22	0.26±0.04	0.11±0.02	2.34 (2.3)	0.019 (0.62)
SS	0.22	0.22	0	0	0	0.22	0.11	0.11	0	0	0.22	0.03±0.02	2.6 (4.96)	0.009 (0.99)
FF	0.11	0.22	0.22	0.22	0	0.22	0	0.33	0.11	0.11	0.19±0.04	0.14±0.05	0.6 (0.43)	0.55 (0.62)
HT	0.44	0.56	0.56	0.11	0.11	0.33	0.44	0.56	0	0	0.44±0.06	0.25±0.96	0.93 (0.97)	0.351 (0.67)

Values shown are standardized scores (*f/n-1*), where *f* represents the number of significant correlations for *neuroserpin* expression between focal brain region and other brain regions and *n* represents the total number of brain regions examined). Statistical comparison of degree centrality between candidate brain regions in bold (Dm, Dl, POA) and other regions in each treatment group are represented in the five right-most columns. Wilcoxon rank sum Z-scores are reported along with effect size (d). Value in parentheses under the p-value is achieved power.

**Figure 5:**
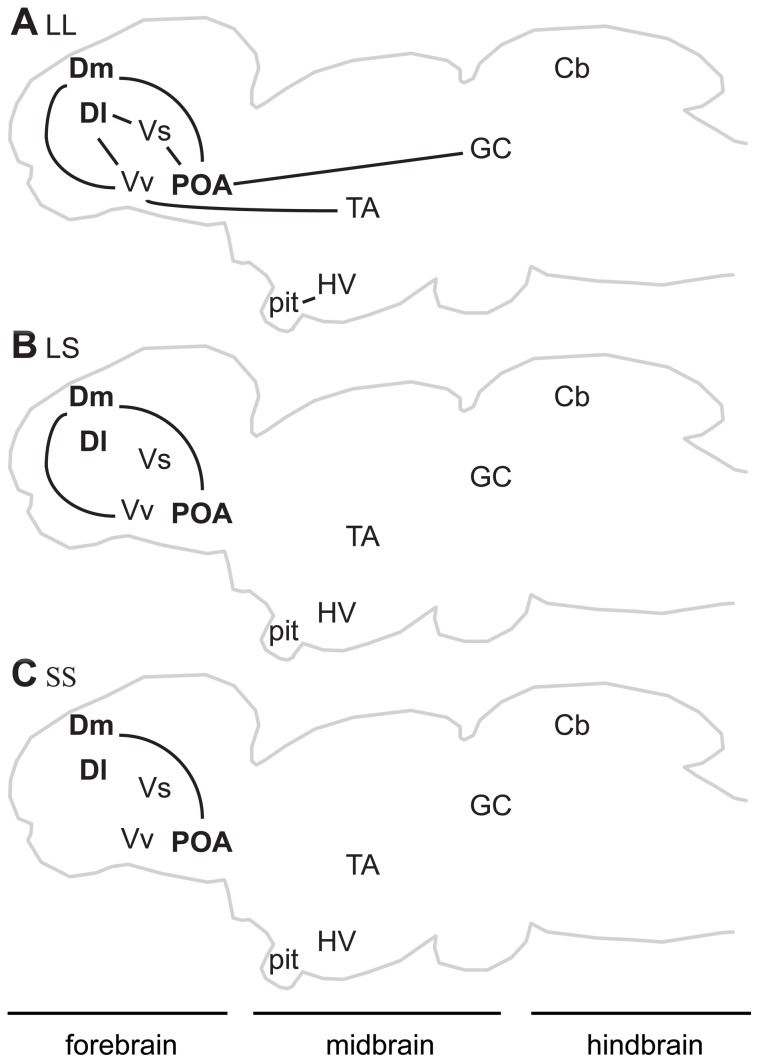
*Neuroserpin* expression network by context. Unique significant positive pairwise correlations relative to FF and HT females in *neuroserpin* expression between brain regions (lines) in A) LL, B) LS, and C) SS exposed females. Brain regions bolded in the schematic sagittal section are those associated with mate preference identified in this study.

To compare across networks we assessed the density of the networks in each male-exposed condition. We observed one unique male exposure correlation that was constant across all three male exposure contexts (Dm with POA) while others appeared only in specific male environments (e.g. Dm with Vv in the presence of large males (LL, LS) but not in small male only conditions (SS), Figure 5). Furthermore, there was a significantly higher network density (i.e. total number of unique significant correlations between regions) for females exposed to LL relative to LS (t = 3.23, p = 0.0026) and SS (t = 3.52, p = 0.0028) male contexts.

## Discussion

Mate preference involves the integration and evaluation of multiple cues from both the external environment and internal physiology. Despite similar behavioral indicators of preference across different social conditions (e.g. females, males, see Figure 1), neuronal phenotypes showed marked context specificity, whether measured by a preference-associated gene (*neuroserpin*) or an IEG (*egr-1*). In male exposed conditions, *neuroserpin* expression was related to mate preference behavior in the putative teleost homologs [55] of the basolateral amygdala (Dm), hippocampus (Dl), and preoptic area (POA) (Figures 2, 3). Of these regions, the POA is an SBN node but Dm and Dl are distinct from previously identified circuits governing sexual response [16], [17]. Our study is the first to show a link between female mate preference behavior and homologs to the basolateral amygdala and hippocampus, suggesting that the integration and evaluation of sensory and reward cues are involved in pre-copulatory mate preference.

The differential gene expression within Dm, Dl, and POA may stem from neural processes regulating a general social preference response rather than a mate choice specific response, however, this is unlikely given that we did not find any significant relationships between preference behavior and *neuroserpin* or *egr-1* expression in the female only (FF) or asocial (AA) conditions (Table S4) despite a similar range of preference behaviors across all social contexts (LL, LS, SS, and FF; see Figure 1). It is also unlikely that our gene expression patterns reflect a general size preference as opposed to mate preference because females preferring a size-matched stimulus (large/large or small/small) showed similar *neuroserpin* expression patterns to those preferring the large male in large/small conditions. This is consistent with our previous results wherein high preference females exhibited comparable whole brain *neuroserpin* and *egr-1* expression levels even if the preferred male was in the small/small condition [30]. Furthermore, specific behavioral components (glides or transits) of the preference score cannot explain our observations, as there were no significant correlations between these behaviors and gene expression in Dm, Dl or the POA (Table S5). Rather, the context-specific significant correlations between gene expression (*neuroserpin* and *egr-1*) and preference behavior suggest that Dm, Dl and the POA are candidate regions associated with processing female mate preference information.

The association between the telencephalic brain regions of Dm and Dl and female preference behavior implies a possible link between sensory processing centers and other regions mediating sexual response (e.g. SBN nodes). Given that in teleosts Dm and Dl receive multimodal input relayed from the preglomerular complex and project to a variety of other fore- and mid-brain regions including the POA [23], [69], these telencephalic brain regions may be prime candidates in mediating sensory integration and discrimination processes that are then directly relayed to the POA or indirectly to the HV to mediate receptivity/copulation behavior. IEG expression within Dm increases with choice behavior as measured by phototaxis in another teleost [70]. The specific functions of the teleost Dm and Dl are still largely unknown, however, lesion studies outside of mate choice contexts have shown that Dm and Dl are involved in analogous measures of emotional and spatial learning in fish, respectively [22]. We acknowledge that we cannot conclusively rule out the involvement of the other regions in female mate preference, as the molecular activity within a brain region associated with female mate preference may be time- and gene-dependent. Examining changes in mate preference behavior after lesioning Dm and/or Dl or other brain regions will be helpful in establishing the regions' causal roles.

Evidence suggests that Dm and Dl are homologs of the tetrapod basolateral amygdala and hippocampus, respectively [17], [55]. As these tetrapod homologs are part of the mesolimbic reward pathway and have been implicated in modulating motivation and reward in rodents [56], [71], [72], female mate preference behavior may also influence or be influenced by this pathway [73]. The hippocampus in females has also been implicated in species recognition and social odor discrimination [74], [75]. The putative reward circuitry in teleosts includes Dm and Dl [17], and Dm, Dl, POA, and HV all express mRNA for dopamine receptors in another teleost [76]. This suggests that reward centers may be involved prior to sexual contact in a mate choice context. Given the putative homology and functional conservation in Dm and Dl between teleost and rodents, we hypothesize that these brain regions could be modulating motivation in female mate preference or arousal behavior, possibly via a homologous mesolimbic reward circuitry in teleosts. Our current results are correlational, therefore future studies should test the functional importance of the mesolimbic reward pathway by pharmacologically manipulating dopamine levels and then measuring any subsequent changes in the strength of female preference.

For many species, female mate choice is an experience-dependent process with females modifying their preference behavior with age (e.g. crickets [77]; bowerbirds [78]; swordtails [27], [79], [80]). Increasing evidence supports a role for learning in mate choice [81], [82] and these experience-dependent behavioral processes require that associated neural circuits be continuously refined and active. *Neuroserpin* and *egr-1* both regulate synaptic plasticity [35]–[37], and previous research has shown that both genes, as well as other markers for synaptic plasticity (e.g. *N-methyl-D-aspartate receptor, neuroligin-3*), are associated with female preference at the whole brain level [30]–[32]. In the current experiment we find positive correlations between *neuroserpin* and *egr-1* expression with preference behavior in brain regions associated with high levels of synaptic plasticity, the putative amygdala and hippocampus regions of the swordtail [83]–[85]. Correlated associations between synaptic plasticity-associated genes and brain regions with mate preference may be important in facilitating the mate evaluation process (e.g. by integrating multiple sensory cues in the putative basolateral amygdala). Similarly dynamic expression patterns within the Dl (putative hippocampus homolog) may mediate recall of specific male phenotypes. Future studies should specifically test the importance of synaptic plasticity in modulating mate choice behavior, either through comparative studies with mate-coercive species or through pharmacological manipulation of synaptic plasticity processes.

Notably, the majority of Social Behavior Network (SBN) nodes that are commonly linked to sexual behavior in other species (e.g. rodents and lizards [15], [18], [54]), did not show correlated expression of *egr-1* or *neuroserpin* with pre-copulatory mate preference behavior in either experiment (*egr-1* and *neuroserpin*). This result was somewhat surprising and suggests the possibility of potential differences in the neural mechanisms underlying mate choice (pre-copulatory assessment) and reproductive (solicitation, sexual receptivity displays, copulation) behavior in some species. Previous research has shown that preference behavior can be independent of reproductive cycle status in *X. nigrensis* females [48], and this behavioral decoupling may be reflected in a reduced role for the SBN nodes in mediating preference behavior. Further, if synaptic plasticity processes are critical in modulating dynamic female assessment of or responses to males, then female preference might be initially regulated by forebrain regions such as Dm and Dl that then coordinate with downstream SBN nodes to initiate receptivity.

It is equally possible, however, that the non-contact nature of our experiment failed to provide the necessary physical cues to elicit rapid SBN activity. Future studies that also include contact trials may help to clarify the relative importance of SBN nodes to Dm and Dl. It is also possible that we did not detect significant correlations in some SBN nodes simply because of the nature of our marker. *Neuroserpin* is associated with synaptic plasticity, and it captured context-specific patterns within brain regions particularly associated with synaptic plasticity. We cannot yet exclude a role for additional SBN nodes in female preference response, and future studies could utilize a different marker to test expression patterns within SBN nodes. Finally, it is also possible that we did not detect more SBN involvement in mate preference due to lack of statistical power in our *egr-1* experiment. IEGs are non-specific markers of neuronal activity, and are frequently used to detect SBN node activity [19], [21], [86], [87]. Ongoing experiments utilizing larger sample sizes to assess IEG expression in females will help to shed light on the relative importance of the SBN in female mate preference.

Variation in behavior can stem from unique changes in gene expression patterns across multiple brain regions [54]. We characterized the network of brain regions expressing *neuroserpin* in response to social stimuli by looking at pair-wise correlations of *neuroserpin* expression between regions in specific social contexts. Candidate regions associated with mate preference (Dm, Dl, and POA) had a significantly higher degree centrality than other regions (Table 3) in each of the male exposed contexts (small/small, large/small, or large/large). Although at different levels of biological organization, studies examining protein interaction networks have found that proteins with a high degree centrality are more essential to the network [88]. This suggests that these regions are important in the *neuroserpin* brain expression network under mate preference conditions.

While the exact function of *neuroserpin* in mate choice remains unknown, it is evident that variation in coordinated expression of *neuroserpin* throughout the brain across male stimuli contexts reflects a neural response that differentiates across male pair compositions. We have proposed a framework wherein our three different male pairings represent a gradient of sensory stimulation and mate choice complexity ranging from most stimulating and complex (LL) to simpler choice environments with less sensory stimulation due to the absence of (SS) or fewer ornamented males engaging in courtship display (LS). Our results suggest that coordinated expression of *neuroserpin* scales with increasing sensory stimulation and complexity of the mate choice conditions (Figure 5). In the minimal choice environment (lacking a large male phenotype, SS), we observed only a single significant correlation (Dm with POA), and this relationship may be due to reciprocal neuroanatomical projections between these regions [69]. However, in the simple mate choice condition (one large male phenotype and one small male phenotype, LS), the number of significant correlations doubled, and in the most complex condition (two large males, LL) we observed eight significant correlations between regions including all three of the candidate preference-specific brain regions (Dm, Dl and POA). As *neuroserpin* is implicated in regulating synaptic plasticity and, in particular, modulating neurite growth [43], the simultaneous assessment of two attractive males (LL) may require refinement of existing neural connections or the establishment of new synaptic connections as females need to assess more information to distinguish between two attractive options. Furthermore, as the LL group provides females with the greatest number of ornamented males engaging in display behavior, it is also possible that increased coordinated *neuroserpin* expression is actually reflecting components of a heightened sensory/physiological response to two good options.

In this study, we begin to identify the network of brain regions associated with mate choice by using both a context specific marker (candidate preference gene) as well as an IEG. This is the first study to identify multisensory processing, spatial learning, and putative reward regions (Dm, Dl) in conjunction with reproductive regions (POA, HV) as putative nodes in a female mate preference pathway. As our study evaluates females in the act of choosing (e.g. presented with two stimuli simultaneously), Dm and Dl may facilitate discernment of stimuli by integrating multi-sensory information prior to enacting a sexual response. Network analysis show that Dm, Dl, POA may be important in mate preference and that correlated patterns of *neuroserpin* expression between regions increase with increasing complexity or sensory stimulation of the mate choice environment.

## Supporting Information

Figure S1 ***In situ*** **hybridization technical controls.** Representative images of antisense (A,C,E) and sense probes (B,D,F) for DIG-labeled *egr-1*, DIG-labeled *neuroserpin*, and S35-labeled *neuroserpin*. S-35 labeled *neuroserpin* images (E & F) are counterstained with cresyl violet.
(TIF)

Figure S2 ***In situ*** **hybridization (ISH) quantification correlations.** (a) Correlation between *neuroserpin* quantification methods on adjacent series. There is a significant positive correlation (r = 0.351, p = 0.008) between optical density measured from digoxigenin ISH and number of *neuroserpin* positive cells measured from S35 ISH. (b) Correlation between *neuroserpin* expression in Dm and preference score using S35 labeled riboprobes. Number of *neuroserpin* positive cells from S35 labeled riboprobes show a significant correlation with preference score (r = 0.405, p = 0.049). Triangles, diamonds, and squares represent LL, LS, and SS exposed females, respectively.
(TIF)

Figure S3 **Gene expression across brain regions in Experiment 1 and 2.** (a) *egr-1* expression and (b) *neuroserpin* expression across the 10 brain regions examined for each group. For Experiment 1 (*egr-1*), colors red, purple, and orange represent LS, FF, and HT, respectively. For Experiment 2 (*neuroserpin*) colors, blue, red, green, purple, and yellow represent LL, LS, SS, FF, and HT, respectively.
(TIF)

Methods S1 **Supplementary materials and methods.**
(DOCX)

Table S1 **Comparisons between** ***in situ*** **hybridization (ISH) quantification methods (mean ± SE) of** ***neuroserpin*** **as related to “high” (> median) and “low” (< median) behavior in Dm.** ** indicates significance after correcting for multiple hypotheses; n.s., not significant.
(DOC)

Table S2 ***Neuroserpin*** **optical density (mean ± SE) comparisons between “high” (> median) and “low” (< median) preference score.** ** indicates significance after correcting for multiple hypotheses; * indicates significance that does not survive multiple hypothesis testing; n.s., not significant.
(DOC)

Table S3 ***Neuroserpin*** **optical density (mean ± SE) comparisons between “high” (> median) and “low” (< median) behaviors.** n.s., not significant.
(DOC)

Table S4 **Correlations between preference score and gene expression in Dm, Dl, POA in non-sexual contexts.**
(DOC)

Table S5 **Correlations between glides, transits and gene expression in Dm, Dl, POA in male exposed environments.**
(DOC)

Table S6 **Correlation between circulating estradiol levels and preference score, glides, transits, and gene expression in different brain regions for each treatment group.**
(DOC)
