# Supplementary figures and images for: Localizing Brain Regions Associated with Female Mate Preference Behavior in a Swordtail
Source: PLoS One. 2012 Nov 29;7(11):e50355. doi: 10.1371/journal.pone.0050355 (PMC3510203; doi:10.1371/journal.pone.0050355)

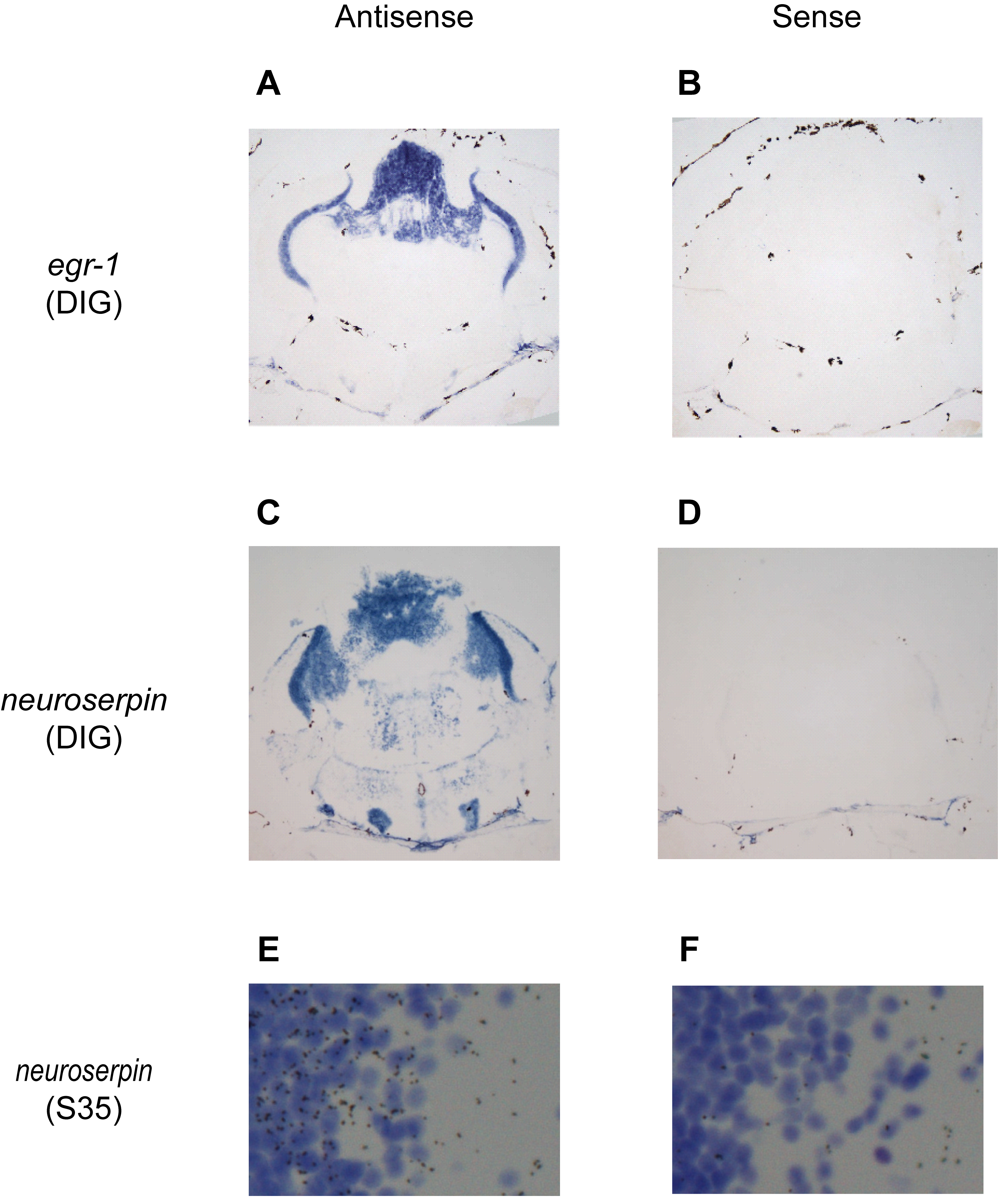

Supplement: Figure S1 — In situ hybridization technical controls. Representative images of antisense (A,C,E) and sense probes (B,D,F) for DIG-labeled egr-1, DIG-labeled neuroserpin, and S35-labeled neuroserpin. S-35 labeled neuroserpin images (E & F) are counterstained with cresyl violet. (TIF) [file pone.0050355.s001.tif]

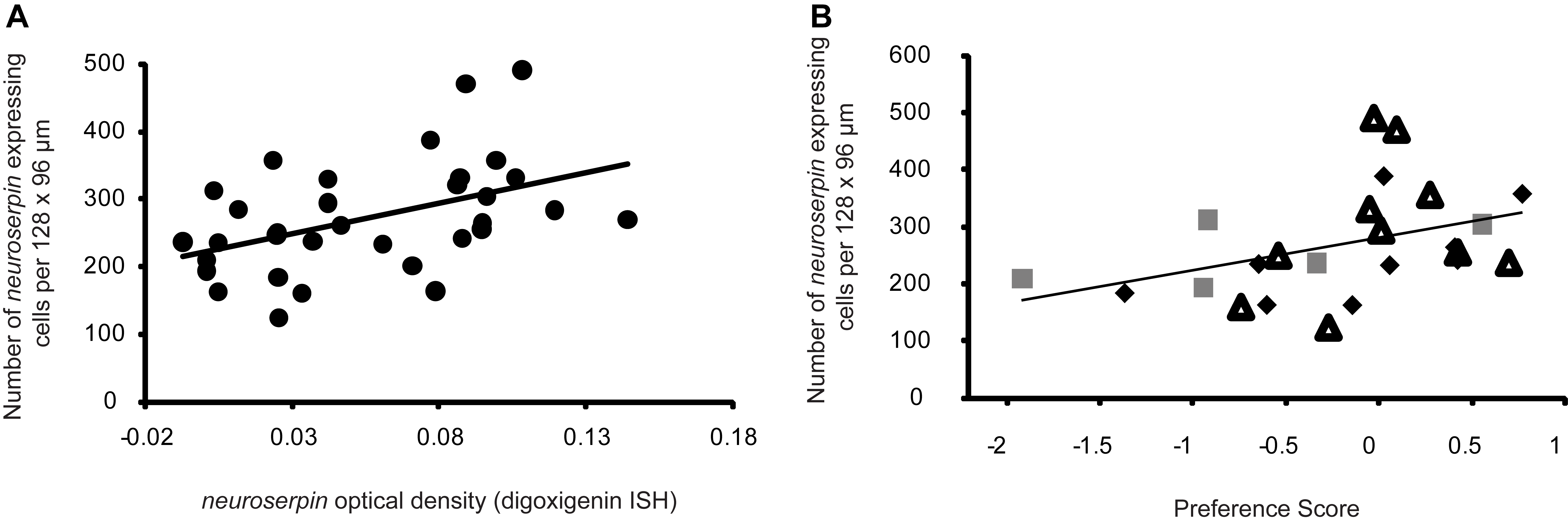

Supplement: Figure S2 — In situ hybridization (ISH) quantification correlations. (a) Correlation between neuroserpin quantification methods on adjacent series. There is a significant positive correlation (r = 0.351, p = 0.008) between optical density measured from digoxigenin ISH and number of neuroserpin positive cells measured from S35 ISH. (b) Correlation between neuroserpin expression in Dm and preference score using S35 labeled riboprobes. Number of neuroserpin positive cells from S35 labeled riboprobes show a significant correlation with preference score (r = 0.405, p = 0.049). Triangles, diamonds, and squares represent LL, LS, and SS exposed females, respectively. (TIF) [file pone.0050355.s002.tif]

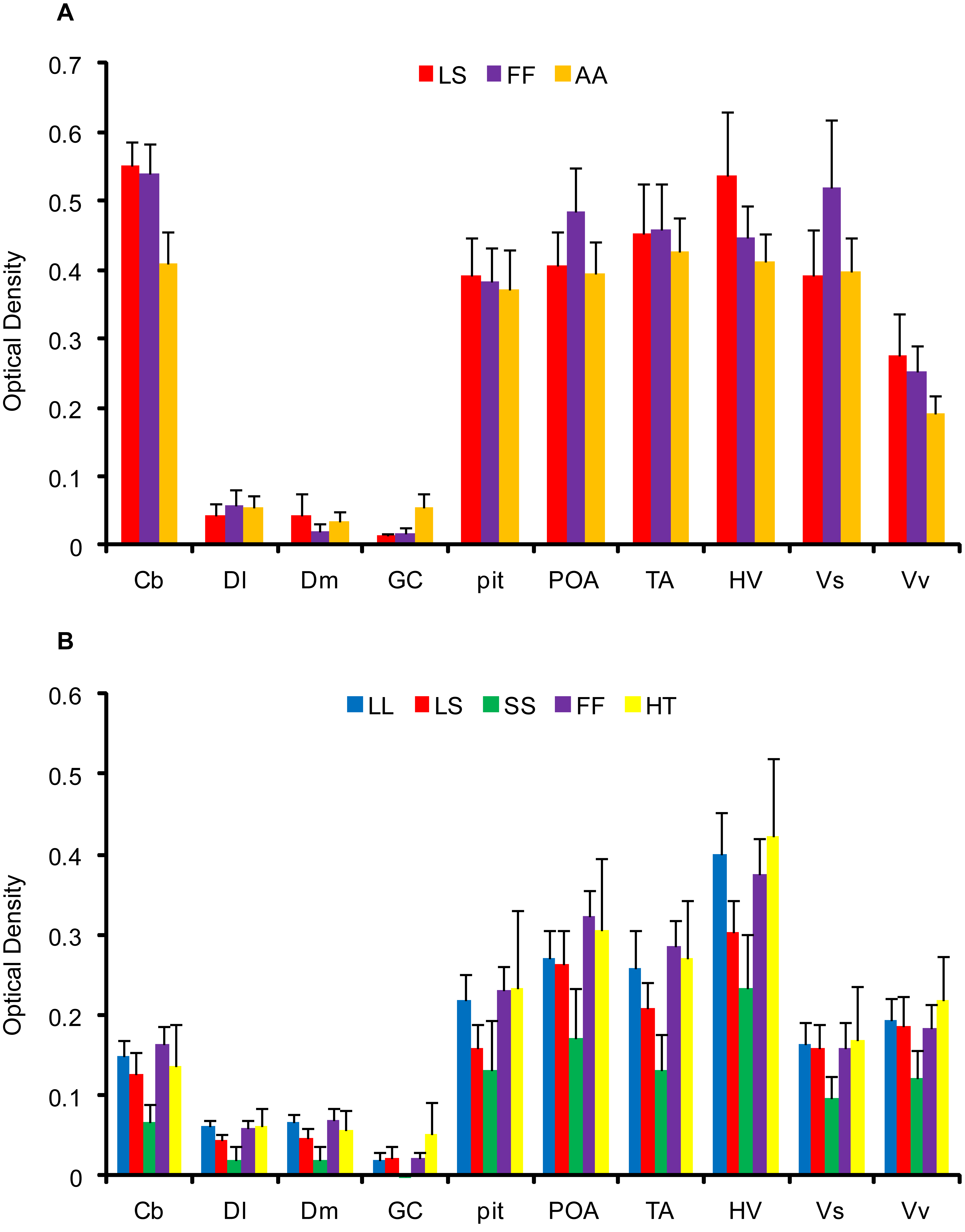

Supplement: Figure S3 — Gene expression across brain regions in Experiment 1 and 2. (a) egr-1 expression and (b) neuroserpin expression across the 10 brain regions examined for each group. For Experiment 1 (egr-1), colors red, purple, and orange represent LS, FF, and HT, respectively. For Experiment 2 (neuroserpin) colors, blue, red, green, purple, and yellow represent LL, LS, SS, FF, and HT, respectively. (TIF) [file pone.0050355.s003.tif]
