# Supplementary material for: Localizing Brain Regions Associated with Female Mate Preference Behavior in a Swordtail
Source: PLoS One. 2012 Nov 29;7(11):e50355. doi: 10.1371/journal.pone.0050355 (PMC3510203; doi:10.1371/journal.pone.0050355)
Supplement: Methods S1 — Supplementary materials and methods. (DOCX) [file pone.0050355.s004.docx]

**Tissue Processing**: Female brains were cryosectioned at 16 µm thickness onto four serial series. For each experiment, all series were simultaneously post-fixed in cold 4% paraformaldahyde/PBS solution, washed in PBS and acetylated in 0.25% acetic anhydride/triethanolamine. Subsequently, slides were washed in 2X standard saline citrate, dehydrated in increasing ethanol series and stored at -80 C. In Experiment 2, one series was used to localize and quantify *neuroserpin* expression using a digoxigenin (DIG) labeled probe (see below) , and an adjacent series was used to validate the use of DIG as a semi-quantitative method by using S35 labeled *neuroserpin* probe (see below).

**Probe Synthesis**: Digoxigenin (DIG) labeled *neuroserpin* and *egr-1* riboprobes were generated using a 1:3 ratio of UTP and DIG-UTP (Roche) following a modified manufacturer’s protocol (Megascript T7/T3, Ambion). A 379 base pair *neuroserpin* DIG probe template was subcloned (Gene Accession #DQ839542.1) by using primer pair 5’- TTATCGCTCTCTGTGGTCTGTGCT -3’and 5’-TGGGCAAATCGGATCACATAGTGG -3’. The 337 base pair *egr-1* probe template was subcloned by using primer pair 5’-CATCCTCCTCATCAACCATCTCG-3’and 5’-TGACTCTGGAAGGGCTTTTGG-3’on the *X. nigrensis* *egr-1* transcript (Gene Accession # DQ835282). After probe synthesis, we removed unincorporated nucleotides via column filtration according to manufacturer’s protocol (Megaclear, Ambion) and checked probe quality through gel electrophoresis.

Radioactive (S35-UTP, 800 Ci/mmol, Perkin Elmer) labeled *neuroserpin* riboprobes were generated following manufacturer’s protocol (Maxiscript T7/T3, Ambion). A 563 base pair probe was subcloned by using primer pair 5’- CAGGCTGAAGTGGAAACTGTGG -3’and 5’- GCATCATCGGTGAAGATTTTGG -3’on the *X. nigrensis* *neuroserpin* transcript (Gene Accession # HM107107). A pilot study showed that this probe template generated a higher signal to noise ratio with S35 UTP than probe template used for the DIG *in situ* hybridization. We used Nucaway spin columns (Ambion) to purify the probe according to manufacturer’s protocol. We verified probe integrity by gel electrophoresis and quantified using a scintillation counter.

***in situ* hybridization**: We used a modified digoxigenin *in situ* hybridization protocol [1]. Briefly, slides were prehybridized with solution containing 50% formamide, 5X SSC, 5X Denhardt’s solution, 250 ug/ml yeast tRNA, and 500 ug/ml herring sperm DNA for 6 hours at 60°C in a hybridization chamber containing chamber buffer solution (50% formamide, 2X SSC). We then hybridized the slides overnight at 65°C with fresh prehybridization solution containing 0.25 ng of antisense riboprobe per slide in hybridization chambers containing buffer. Following hybridization we conducted washes at low stringency (two washes in 2X SSC at room temperature), then RNase A treated the slides (0.5M NaCl, 10 mM Tris pH 8.0, 2.25 mM EDTA, 0.2 µg/ml RNase A), followed by increasingly stringent washes (2X, 1X, 0.5X, 0.25X SSC) and then a final wash in Buffer B1 (100 mM Tris pH 7.5, 150 mM NaCl). Sections were then incubated overnight at 4°C with Anti-Digoxigenin AP antibody (Roche). After antibody incubation we washed sections twice in Buffer B1 and then blocked endogenous alkaline phosphatase activity with a 30 minutes wash in the dark in Buffer B3 (100mM Tris pH 9.5, 100 mM NaCl, 50 mM MgCl2, 5 mM levamisole). We carried out colorimetric detection using NBT/BCIP stock solution (Roche). We stopped the colorimetric reaction (75 minutes for *neuroserpin* and 95 minutes for *egr-1* detection) by rinsing sections three times in ultrapure type 1 water, then progressively dehydrated sections in ethanol washes (25%, 50%, 70%, 95%), and finally coverslipped the slides with Permount adhesive (Fisher). Within each experiment all individuals were processed simultaneously to avoid any potential colorimetric development differences across individuals due to batch effects. Both DIG labeled *egr-1* and *neuroserpin* sense riboprobes showed negligible or no expression (Fig. S1).

To validate our use of DIG-labeled probes as a semi-quantitative method, we also conducted an *in situ* hybridization using S35 *neuroserpin* riboprobes on an adjacent series to the DIG-labeled series of Experiment 2, by modifying a previous protocol [2]. Briefly, for all slides we applied 250 µl of hybridization solution (50% formamide, 10% Dextran sulfate, 300 mM sodium chloride, 8 mM Tris pH 8.0, 0.8 mM EDTA pH 8.0, 1X Denhardt’s solution, 8 mM dithiothreitol (DTT), 250 µg/mL yeast tRNA, and 50 µg/mL Herring Sperm DNA) to each slide with 4500 CPM of probe/µl of solution, coverslipped and hybridized 16 hours at 65°C in hybridization chambers with buffer (50% formamide, 2X SSC). After hybridization, we washed slides to remove nonspecific probe binding as follows: 1 hour in 4X SSC at 65 C, 1 hour in 50% formamide and 2X SSC, 15 minutes in RNAse buffer (0.5M NaCl, 10mM Tris pH 8.0, 0.1125 µM EDTA) 30 minutes RNAse treatment (20 µg/ml) at 37°C, 15 minutes in RNAse buffer, 15 minutes in 2X SSC and finally 15 minutes in 0.1X SSC. All washes unless otherwise stated occurred at room temperature and contained 1 mM DTT except for the RNAse treatment. After washes, slides were dehydrated in increasing ethanol series (25%, 50%, 70%, 95%, 100%) containing 300 mM ammonium acetate and cleared in xylenes. To visualize the probes, all slides were dipped in 37ºC Kodak NTB2 emulsion (Kodak) and dried at 60ºC for 2 hours. Slides were then stored in light proof boxes at 4ºC for 7 days. We developed slides using D19 developer (Kodak) and Kodak Fixer (Kodak). Afterwards, we stained the tissue in cresyl violet and coverslipped with Permount (Fisher).Sense S35-labeled probes showed nearly no signal above background (Figure S1).

**Brain Regions Analyzed:** Brain section images were captured at 4X using a Nikon 12-bit 2-megapixel monochrome camera (DS-2MBWc). For each brain region, we used Nikon NIS Elements 2.3 to measure a standardized rectangular box (area of box given after each brain region, see below) within the borders of each brain region and measured the mean intensity of *neuroserpin* and *egr-1* expression within the box. Unless otherwise stated, the measuring box was always placed in the middle of the brain region on the dorsal-ventral plane. We measured the mean intensity in both hemispheres if available and then averaged the values for the section. For all brain regions we then proceeded to measure the mean intensity for all sections of the individual in which we could identify the desired brain region and then averaged for that individual. Depending on the size of the brain region, the number of sections averaged per individual ranged from two to eight consecutive sections. Consecutive sections spanned 48 μm apart. Specifically, we measured Dl (19906 μm^2^) & Dm (19906 μm^2^) for sections from the rostral most section until the disappearance of the anterior commissure; Vv (5877 μm^2^) & Vs (5877 μm^2^) for sections only containing the telencephalic ventricle; POA (2843 μm^2^) for sections with anterior commissure present until the appearance of the optic tectum; Pit (12832 μm^2^) for all sections observed; HV (1737 μm^2^) from the pituitary until the lateral extension of the lateral hypothalamus; TA (5339 μm^2^) for 3 sections preceding the lateral extension of the dorsal hypothalamus; Cb (29152 μm^2^) and CG (7298 μm^2^) for all sections observed (see main text for brain region abbreviations). All image capture and data collection was done by a single person blind to the treatments.

**References:**

1. Shoemaker CM, Queen J, Crews D (2007) Response of candidate sex-determining genes to changes in temperature reveals their involvement in the molecular network underlying temperature dependent sex determination. Molecular Endocrinology 21: 2750-2763.

2. Hoke KL, Burmeister SS, Fernald RD, Rand AS, Ryan MJ, et al. (2004) Functional mapping of the auditory midbrain during mate call reception. J Neurosci 24: 11264-11272.
