# Supplementary material for: Localizing Brain Regions Associated with Female Mate Preference Behavior in a Swordtail
Source: PLoS One. 2012 Nov 29;7(11):e50355. doi: 10.1371/journal.pone.0050355 (PMC3510203; doi:10.1371/journal.pone.0050355)
Supplement: Table S1 — Comparisons between in situ hybridization (ISH) quantification methods (mean ± SE) of neuroserpin as related to “high” (> median) and “low” (< median) behavior in Dm. ** indicates significance after correcting for multiple hypotheses; n.s., not significant. (DOC) [file pone.0050355.s005.doc]

Table S1. Comparisons between *in situ* hybridization (ISH) quantification methods (mean ± SE) of *neuroserpin* as related to “high” (> median) and “low” (< median) behavior in Dm.

|  | Male Exposed (LL, LS, and SS) | | | | | | | Female Exposed (FF) | | | | | | |
| --- | --- | --- | --- | --- | --- | --- | --- | --- | --- | --- | --- | --- | --- | --- |
|  | Digoxigenin ISH  (Optical Density) | | | S35 ISH (# of *neuroserpin* positive cells) | | | Consistent between methods? | Digoxigenin ISH (Optical Density) | | | S35 ISH (# of *neuroserpin* positive cells) | | | Consistent between methods? |
|  | High | Low | p-value | High | Low | p-value |  | High | Low | p-value | High | Low | p-value |  |
| Preference Score | 0.068 ± 0.008 | 0.025 ± 0.009 | ******  (0.003) | 309.56 ± 22.85 | 213.89 ± 18.04 | ******  (0.003) | Yes | 0.079 ± 0.016 | 0.075 ± 0.026 | **n.s.**  (0.889) | 287.71 ± 30.94 | 274.75 ± 16.14 | **n.s.**  (0.723) | Yes |
| Transits | 0.04  ± 0.013 | 0.06  ± 0.009 | **n.s.**  (0.239) | 279.33 ± 31.33 | 268.24 ± 23.69 | **n.s.**  (0.783) | Yes | 0.046 ± 0.009 | 0.082 ± 0.027 | **n.s.**  (0.255) | 259.95 ± 26.61 | 302.97 ± 15.16 | **n.s.**  (0.209) | Yes |
| Glides | 0.065 ± 0.011 | 0.035 ±  0.01 | **n.s.**  (0.072) | 303.04 ± 32.09 | 245.43 ± 22.10 | **n.s.**  (0.148) | Yes | 0.046 ± 0.009 | 0.082 ± 0.027 | **n.s.**  (0.255) | 259.95 ± 26.61 | 302.97 ± 15.16 | **n.s.**  (0.209) | Yes |
| Association Bias | 0.05  ± 0.011 | 0.045 ± 0.012 | **n.s.**  (0.761) | 256.5 ± 19.36 | 292.85 ± 35.12 | **n.s.**  (0.364) | Yes | 0.098 ± 0.022 | 0.054 ± 0.016 | **n.s.**  (0.156) | 304.85 ± 16.42 | 267.15 ± 25.06 | **n.s.**  (0.255) | Yes |

** indicates significance after correcting for multiple hypotheses; n.s., not significant.
