# Supplementary material for: Localizing Brain Regions Associated with Female Mate Preference Behavior in a Swordtail
Source: PLoS One. 2012 Nov 29;7(11):e50355. doi: 10.1371/journal.pone.0050355 (PMC3510203; doi:10.1371/journal.pone.0050355)
Supplement: Table S2 — Neuroserpin optical density (mean ± SE) comparisons between “high” (> median) and “low” (< median) preference score. ** indicates significance after correcting for multiple hypotheses; * indicates significance that does not survive multiple hypothesis testing; n.s., not significant. (DOC) [file pone.0050355.s006.doc]

Table S2. *Neuroserpin* optical density (mean ± SE) comparisons between “high” (> median) and “low” (< median) preference score.

| Brain Region | Male Exposed (LL, LS, and SS) | | | | Female Exposed (FF) | | | |
| --- | --- | --- | --- | --- | --- | --- | --- | --- |
|  | Preference Score | | | | Preference Score | | | |
|  | High | Low | t-value | p-value | High | Low | t-value | p-value |
| Dm | 0.068 ± 0.008 | 0.025 ± 0.009 | 3.284 | ******(0.003) | 0.079 ± 0.016 | 0.075 ± 0.026 | 0.144 | **n.s.** (0.889) |
| Dl | 0.059 ± 0.006 | 0.028 ± 0.008 | 2.91 | ******(0.008) | 0.065 ± 0.014 | 0.061 ± 0.019 | 0.169 | **n.s.** (0.87) |
| Cb | 0.136 ± 0.02 | 0.104 ± 0.023 | 1.02 | **n.s.** (0.318) | 0.164 ± 0.0389 | 0.18 ± 0.027 | -0.342 | **n.s.** (0.87) |
| GC | 0.02 ± 0.01 | 0.015 ± 0.01 | 0.298 | **n.s.** (0.768) | 0.026 ± 0.013 | 0.02 ± 0.015 | 0.262 | **n.s.** (0.801) |
| Pit | 0.209 ± 0.026 | 0.134 ± 0.032 | 1.834 | **n.s.** (0.08) | 0.252 ± 0.028 | 0.222 ± 0.066 | 0.415 | **n.s.** (0.692) |
| POA | 0.313 ± 0.03 | 0.172 ± 0.029 | 3.291 | ****** (0.003) | 0.292 ± 0.04 | 0.378 ± 0.04 | -1.424 | **n.s.** (0.204) |
| TA | 0.233 ± 0.034 | 0.17 ± 0.031 | 1.359 | **n.s.** (0.188) | 0.281 ± 0.044 | 0.313 ± 0.056 | -0.443 | **n.s.** (0.673) |
| HV | 0.385 ± 0.039 | 0.248 ± 0.037 | 2.489 | ***** (0.02) | 0.393 ± 0.056 | 0.401 ± 0.075 | -0.076 | **n.s.** (0.941) |
| Vs | 0.162 ± 0.021 | 0.125 ± 0.026 | 1.084 | **n.s.** (0.289) | 0.183 ± 0.069 | 0.17 ± 0.027 | 0.186 | **n.s.** (0.859) |
| Vv | 0.186 ±0.026 | 0.151 ± 0.029 | 0.881 | **n.s.** (0.388) | 0.171 ± 0.059 | 0.218 ± 0.035 | -0.718 | **n.s.** (0.504) |

** indicates significance after correcting for multiple hypotheses; * indicates significance that does not survive multiple hypothesis testing; n.s., not significant.
