# Supplementary material for: Localizing Brain Regions Associated with Female Mate Preference Behavior in a Swordtail
Source: PLoS One. 2012 Nov 29;7(11):e50355. doi: 10.1371/journal.pone.0050355 (PMC3510203; doi:10.1371/journal.pone.0050355)
Supplement: Table S3 — Neuroserpin optical density (mean ± SE) comparisons between “high” (> median) and “low” (< median) behaviors. n.s., not significant. (DOC) [file pone.0050355.s007.doc]

Table S3. *Neuroserpin* optical density (mean ± SE) comparisons between “high” (> median) and “low” (< median) behaviors.

| Brain Region | Male Exposed (LL, LS, and SS) | | | | | | | | Female Exposed (FF) | | | | | | | |
| --- | --- | --- | --- | --- | --- | --- | --- | --- | --- | --- | --- | --- | --- | --- | --- | --- |
|  | Glides | | | | Transits | | | | Glides | | | | Transits | | | |
|  | High | Low | t-value | p-value | High | Low | t-value | p-value | High | Low | t-value | p-value | High | Low | t-value | p-value |
| Dm | 0.065 ± 0.011 | 0.035 ± 0.01 | 1.88 | **n.s.**  (0.072) | 0.04 ± 0.013 | 0.06 ± 0.009 | -1.2 | **n.s.**  (0.239) | 0.046 ± 0.009 | 0.082 ± 0.027 | -1.25 | **n.s.**  (0.255) | 0.046 ± 0.009 | 0.082 ± 0.027 | -1.25 | **n.s.**  (0.255) |
| Dl | 0.056 ± 0.009 | 0.036 ± 0.008 | 1.57 | **n.s.**  (0.128) | 0.04 ± 0.01 | 0.053 ± 0.007 | -1.03 | **n.s.**  (0.31) | 0.04 ± 0.011 | 0.068 ± 0.017 | -1.31 | **n.s.**  (0.236) | 0.04 ± 0.011 | 0.068 ± 0.017 | -1.31 | **n.s.**  (0.236) |
| Cb | 0.133 ± 0.023 | 0.116 ± 0.022 | 0.53 | **n.s.**  (0.598) | 0.124 ± 0.22 | 0.132 ± 0.02 | -0.25 | **n.s.**  (0.799) | 0.161 ± 0.022 | 0.146 ± 0.04 | 0.32 | **n.s.**  (0.756) | 0.161 ± 0.022 | 0.146 ± 0.04 | 0.32 | **n.s.**  (0.756) |
| GC | 0.011 ± 0.008 | 0.024 ± 0.011 | -0.89 | **n.s.**  (0.381) | 0.01 ± 0.008 | 0.025 ± 0.011 | -1.09 | **n.s.**  (0.285) | 0.013 ± 0.01 | 0.02 ± 0.016 | -0.39 | **n.s.**  (0.706) | 0.013 ± 0.01 | 0.02 ± 0.016 | -0.39 | **n.s.**  (0.706) |
| Pit | 0.203 ± 0.031 | 0.158 ± 0.03 | 1.02 | **n.s.**  (0.315) | 0.16 ± 0.039 | 0.2 ± 0.023 | -0.9 | **n.s.**  (0.374) | 0.186 ± 0.057 | 0.269 ± 0.033 | -1.23 | **n.s.**  (0.261) | 0.186 ± 0.057 | 0.269 ± 0.033 | -1.23 | **n.s.**  (0.261) |
| POA | 0.281 ± 0.037 | 0.212 ± 0.034 | 1.35 | **n.s.**  (0.188) | 0.226 ± 0.042 | 0.278 ± 0.029 | -1.0 | **n.s.**  (0.324) | 0.293 ± 0.031 | 0.343 ± 0.062 | -0.71 | **n.s.**  (0.502) | 0.293 ± 0.031 | 0.343 ± 0.062 | -0.71 | **n.s.**  (0.502) |
| TA | 0.245 ± 0.04 | 0.173 ± 0.034 | 1.39 | **n.s.**  (0.176) | 0.221 ± 0.042 | 0.21 ± 0.03 | 0.2 | **n.s.**  (0.837) | 0.299 ± 0.062 | 0.291 ± 0.041 | 0.1 | **n.s.**  (0.919) | 0.299 ± 0.062 | 0.291 ± 0.041 | 0.1 | **n.s.**  (0.919) |
| HV | 0.363 ± 0.046 | 0.293 ± 0.044 | 1.07 | **n.s.**  (0.295) | 0.316 ± 0.05 | 0.35 ± 0.42 | -0.52 | **n.s.**  (0.605) | 0.32 ± 0.053 | 0.4 ± 0.08 | -0.83 | **n.s.**  (0.435) | 0.32 ± 0.053 | 0.4 ± 0.08 | -0.83 | **n.s.**  (0.435) |
| Vs | 0.147 ± 0.017 | 0.151 ± 0.031 | -0.11 | **n.s.**  (0.912) | 0.132 ± 0.02 | 0.17 ± 0.028 | -1.09 | **n.s.**  (0.283) | 0.165 ± 0.049 | 0.152 ± 0.045 | 0.19 | **n.s.**  (0.849) | 0.165 ± 0.049 | 0.152 ± 0.045 | 0.19 | **n.s.**  (0.849) |
| Vv | 0.189 ± 0.028 | 0.165 ± 0.03 | 0.57 | **n.s.**  (0.574) | 0.172 ± 0.03 | 0.185 ± 0.28 | -0.31 | **n.s.**  (0.752) | 0.2 ± 0.042 | 0.166 ± 0.048 | 0.54 | **n.s.**  (0.604) | 0.2 ± 0.042 | 0.166 ± 0.048 | 0.54 | **n.s.**  (0.604) |

n.s., not significant.
