# Supplementary material for: Localizing Brain Regions Associated with Female Mate Preference Behavior in a Swordtail
Source: PLoS One. 2012 Nov 29;7(11):e50355. doi: 10.1371/journal.pone.0050355 (PMC3510203; doi:10.1371/journal.pone.0050355)
Supplement: Table S4 — Correlations between preference score and gene expression in Dm, Dl, POA in non-sexual contexts. (DOC) [file pone.0050355.s008.doc]

Table S4. Correlations between preference score and gene expression in Dm, Dl, POA in non-sexual contexts.

|  | Experiment 1  (*egr-1)* | | | | Experiment 2  (*neuroserpin*) | |
| --- | --- | --- | --- | --- | --- | --- |
|  | FF | | AA | | FF | |
|  | Correlation coefficient | p-value | Correlation coefficient | p-value | Correlation coefficient | p-value |
| Preference Score | | | | | | |
| Dm | 0.301 | 0.511 | -0.239 | 0.505 | 0.239 | 0.536 |
| Dl | 0.329 | 0.471 | -0.265 | 0.458 | 0.183 | 0.636 |
| POA | -0.191 | 0.711 | -0.43 | 0.247 | -0.308 | 0.418 |
