# Supplementary material for: Localizing Brain Regions Associated with Female Mate Preference Behavior in a Swordtail
Source: PLoS One. 2012 Nov 29;7(11):e50355. doi: 10.1371/journal.pone.0050355 (PMC3510203; doi:10.1371/journal.pone.0050355)
Supplement: Table S5 — Correlations between glides, transits and gene expression in Dm, Dl, POA in male exposed environments. (DOC) [file pone.0050355.s009.doc]

Table S5. Correlations between glides, transits and gene expression in Dm, Dl, POA in male exposed environments.

|  | Experiment 1  LS  (*egr-1)* | | Experiment 2  LL, LS and SS  (*neuroserpin*) | |
| --- | --- | --- | --- | --- |
|  | Correlation coefficient | p-value | Correlation coefficient | p-value |
| Glides | | | | |
| Dm | 0.714 | 0.11 | 0.204 | 0.327 |
| Dl | 0.771 | 0.072 | 0.244 | 0.239 |
| POA | -0.257 | 0.622 | 0.164 | 0.432 |
| Transits | | | | |
| Dm | 0.257 | 0.622 | -0.306 | 0.136 |
| Dl | 0.314 | 0.544 | -0.258 | 0.213 |
| POA | -0.257 | 0.622 | -0.324 | 0.113 |
