# Supplementary material for: Localizing Brain Regions Associated with Female Mate Preference Behavior in a Swordtail
Source: PLoS One. 2012 Nov 29;7(11):e50355. doi: 10.1371/journal.pone.0050355 (PMC3510203; doi:10.1371/journal.pone.0050355)
Supplement: Table S6 — Correlation between circulating estradiol levels and preference score, glides, transits, and gene expression in different brain regions for each treatment group. (DOC) [file pone.0050355.s010.doc]

Table S6. Correlation between circulating estradiol levels and preference score, glides, transits, and gene expression in different brain regions for each treatment group.

|  | Experiment 1  (*egr-1*) | | | | | | Experiment 2  (*neuroserpin*) | | | |
| --- | --- | --- | --- | --- | --- | --- | --- | --- | --- | --- |
|  | LS | | FF | | AA | | LL, LS and SS | | FF | |
|  | Correlation coefficient | p-value | Correlation coefficient | p-value | Correlation coefficient | p-value | Correlation coefficient | p-value | Correlation coefficient | p-value |
| Preference Score | -0.024 | 0.957 | -0.275 | 0.509 | -0.607 | 0.062 | -0.296 | 0.149 | -0.692 | 0.067 |
| Glides | -0.071 | 0.879 | -0.54 | 0.166 | -0.343 | 0.331 | 0.401 | 0.047 | 0.636 | 0.065 |
| Transits | -0.07 | 0.879 | -0.18 | 0.668 | 0.557 | 0.093 | 0.37 | 0.06 | 0.726 | 0.026 |
| Dm | -0.709 | 0.114 | -0.513 | 0.238 | 0.152 | 0.674 | -0.106 | 0.611 | 0.033 | 0.932 |
| Dl | -0.649 | 0.162 | -0.317 | 0.488 | 0.139 | 0.701 | -0.133 | 0.525 | -0.1017 | 0.794 |
| Cb | 0.543 | 0.207 | 0.207 | 0.621 | 0.432 | 0.212 | -0.139 | 0.505 | -0.015 | 0.967 |
| GC | 0.02 | 0.971 | 0.081 | 0.862 | 0.209 | 0.588 | -0.166 | 0.427 | -0.29 | 0.448 |
| Pit | 0.646 | 0.116 | -0.262 | 0.53 | 0.667 | 0.049 | -0.327 | 0.117 | -0.377 | 0.316 |
| POA | 0.184 | 0.725 | 0.398 | 0.433 | 0.5 | 0.169 | -0.077 | 0.712 | 0.138 | 0.722 |
| TA | 0.805 | 0.028 | 0.334 | 0.417 | 0.484 | 0.155 | -0.129 | 0.545 | -0.129 | 0.739 |
| HV | 0.714 | 0.071 | 0.123 | 0.771 | 0.69 | 0.027 | -0.057 | 0.785 | -0.147 | 0.705 |
| Vs | 0.329 | 0.523 | -0.626 | 0.183 | 0.454 | 0.219 | -0.173 | 0.406 | 0.316 | 0.444 |
| Vv | -0.391 | 0.443 | -0.666 | 0.101 | 0.275 | 0.44 | -0.109 | 0.611 | 0.654 | 0.078 |
